# Dietary Salt Disrupts Tricarboxylic Acid Cycle and Induces Tau Hyperphosphorylation and Synapse Dysfunction during Aging

**DOI:** 10.14336/AD.2022.0220

**Published:** 2022-10-01

**Authors:** Minghao Yuan, Yangyang Wang, Jie Wen, Feng Jing, Qian Zou, Yinshuang Pu, Tingyu Pan, Zhiyou Cai

**Affiliations:** ^1^Chongqing Medical University, Chongqing, China.; ^2^Department of Neurology, Chongqing School, University of Chinese Academy of Sciences, Chongqing, China.; ^3^Department of Neurology, Chongqing General Hospital, Chongqing, China.; ^4^Chongqing Key Laboratory of Neurodegenerative Diseases, Chongqing, China.; ^5^Guangdong Medical University, Guangdong, China.

**Keywords:** Dietary Salt, Tricarboxylic Acid Cycle, Tau Hyperphosphorylation, Synapse dysfunction, Cognitive Impairment

## Abstract

Dietary salt causes synaptic deficits and tau hyperphosphorylation, which are detrimental to cognitive function. However, the specific effects of a high-salt diet on synapse and tau protein remain poorly understood. In this study, aged (15-month-old) C57BL/6 mice received a normal (0.5% NaCl) or high-salt (8% NaCl) diet for 3 months, and N2a cells were treated with normal culture medium or a NaCl medium (40 mM). Spatial learning and memory abilities were tested using the Morris water maze. The levels of metabolites and related enzymes in the tricarboxylic acid (TCA) cycle were confirmed using liquid chromatography-tandem mass spectrometry, western blotting, and immunofluorescence. We also investigated synapse morphology and the phosphorylation of tau protein. Under the high-salt diet, mice displayed impaired learning and memory compared to mice fed the normal diet. Furthermore, excessive salt intake disturbed the TCA cycle in both animals and cells compared to the respective normal controls. High dietary salt reduced postsynaptic density protein 95 (PSD95) and brain-derived neurotrophic factor (BDNF) expression, impaired neurons, and caused synaptic loss in the mice. We also detected tau hyperphosphorylation at different sites (Thr205, Thr231, and Thr181) without increasing total tau levels in response to high salt treatment, both in vivo and in vitro. We concluded that elevated salt intake impairs the TCA cycle and induces tau hyperphosphorylation and synapse dysfunction during aging, which ultimately results in cognitive impairment.

Salt-rich diets are common worldwide and increasingly considered unhealthy, being an independent risk factor for hypertension [1], stroke [2], cerebral small vessel disease [3], and cognitive impairment [4, 5]. The average global dietary salt intake is 12 g per day, more than 2.5-fold the recommended intake (1 g per day) [6]. A recent study showed that a salt-rich diet induces tau hyperphosphorylation (Ser202, Thr205, and Thr231) without increasing total tau levels in mice [7]. In addition, a high-salt diet impairs long- and short-term memory and reduces the expression of enzymes linked to the regulation of synaptic plasticity, including synaptophysin (Syn), calmodulin-dependent kinase II (CaMK-II), CaMK-IV, and extracellular signal-regulated kinase 1/2, in mice [8]. Tau hyperphosphorylation and synaptic dysfunction are generally considered the major pathological changes causing cognitive impairment in patients with Alzheimer’s disease [9, 10].

The tricarboxylic acid (TCA) cycle, also known as the Krebs cycle, is the final metabolic pathway of glucose, lipids, and amino acids. Previous research on the kidney has implied that a chronic high-salt diet inhibits or decreases the activities of enzymes involved in the TCA cycle, such as aconitase, fumarase, malate dehydrogenase, isocitrate dehydrogenase, and pyruvate carboxylase [11]. However, little is known regarding how such a diet affects the TCA cycle in the brain. In general, damage to the TCA cycle causes enzyme accumulation or depletion and an uneven distribution of intermediate products, eventually resulting in energy deficit. The TCA cycle disruption appear to be linked to synaptic dysfunction and tau phosphorylation. For instance, a study on astrocytes showed that inhibiting aconitase increases tau phosphorylation and decreases the expression of synaptic-related proteins, specifically glutamate receptor 2, postsynaptic density protein 93 (PSD93), and PSD95 [12]. Moreover, isocitrate is significantly associated with phospho-tau (Thr181) in the plasma and cerebrospinal fluid of patients with Alzheimer’s disease [13]. Finally, decreased α-ketoglutarate dehydrogenase activity lowers ATP production, and chronic ATP deficiency may lead to tau hyperphosphorylation [14]. However, how the TCA cycle, tau hyperphosphorylation, and synaptic dysfunction are related is not fully understood. Based on the previous studies [11-14], we hypothesized that dietary salt leads to a “broken” TCA cycle, which potentially plays a role in inducing tau hyper-phosphorylation and synapse dysfunction, culminating in cognitive impairment. The aim of our study was to define the effects of dietary salt on the TCA cycle, synapse, and tau hyperphosphorylation. We also hoped to gain a better understanding of the potential association between the TCA cycle and salt-induced synapse dysfunction or tau hyperphosphorylation, which would have an impact on future research in this field.

## MATERIAL AND METHODS

### Animals and treatments

Aged male C57BL/6 mice (15 months old, weighing 25-33 g) were purchased from Chengdu Dossy Experimental Animals Co., Ltd. (Chengdu, China) and housed in standard cages in a temperature-controlled facility with 12 h light/dark cycle. All animal experiments were approved by the Research Ethics Committee of Chongqing Medical University. The experimental procedures followed the National Institutes of Health Guide for the Care and Use of Laboratory Animals. Mice received tap water ad libitum and normal (0.4% NaCl, normal diet, ND) or sodium-rich (8% NaCl, high salt diet, HSD) chow (Tengxin Biotechnology Co., Ltd., Chongqing, China) for 12 weeks [7]. Thereafter, C57BL/6 mice (18 months old) were tested in the Morris water maze (MWM). Following testing, the mice were euthanized via decapitation for liquid chromatography-tandem mass spectrometry (LC-MS/MS), transmission electron microscopy (TEM), and western blotting analysis, to avoid indirect effects on tau phosphorylation caused by the hypothermic condition [15], or anesthesia for immunofluorescence staining. After euthanasia, the brain was immediately removed, washed with ice-cold normal saline, and stored at -80°C or 4°C for further analyses.

### Cell culture and treatments

The mouse neuroblastoma cell line Neuro-2a (N2a) was purchased from Procell Life Science & Technology Co., Ltd. (Wuhan, China). The N2a cells were cultured in Dulbecco’s modified Eagle’s medium nutrient mixture F-12 (DMEM-F12, Gibco, Carlsbad, CA, USA) supplemented with 10% fetal bovine serum (FBS; Invitrogen, Irvine, CA, USA) and antibiotics (100 U/mL penicillin and 100 μg/mL streptomycin), and then incubated in a humidified atmosphere with 5% CO_2_ at 37°C. The high-salt N2a cell model was established by treating them with NaCl medium (containing an additional 40 mM NaCl) for 24 h [16] (NaCl group). Non-treated N2a cells were placed into a control (CON) group.

### MWM

Spatial learning and memory were evaluated using the MWM, initiated after 3 months of HSD. To set up the maze, a circular platform (8 cm in diameter) was placed 1 cm below the water surface in the northeast quadrant of a round pool (1.2 m in diameter and 0.4 m in depth, 25 ± 1°C). The maze, which was not visible, was divided into four equal quadrants (Ⅰ, Ⅱ, Ⅲ, Ⅳ). A camera was located above the center of the maze to record the movement of the mice and send the data to ANY-maze video tracking software (Stoelting Co., Wood Dale, IL, USA).

Mice (n=15/group) that were adapted to the experimental environment were trained 4 times per day for 5 consecutive days as acquisition trials. The targeted platform was placed in the center of quadrant Ⅲ, and the mice were placed in a different quadrant and allowed to swim freely for 60 s. Upon finding the targeted platform, mice were allowed to stay on the platform for 5 s before being removed, wiped dry, and placed back in their cages. If any mice failed to find the platform within 60 s, they were picked up and placed on the platform for 30 s. On the day following acquisition testing (day 6), an expeditionary test without the platform was performed. Mice were placed in the quadrant opposite the northeast quadrant, and allowed to swim and search for the platform for 60 s. All data were recorded by the ANY-maze video tracking software (Stoelting Co., Wood Dale, USA), including total swimming duration and distance, swimming duration and distance in the quadrants, and the number of times the mice passed through the targeted platform location within 60 s. The observer was blinded to the experimental conditions while performing the MWM.

### LC-MS/MS

#### Extraction of metabolites

Brain tissues (n=5/group) were homogenized and extracted with 1 mL of cold methanol/acetonitrile/H_2_O (2:2:1, v/v/v) and then sonicated at a low temperature (twice for 30 min each). After storing the samples at -20 °C for 1 h, they were centrifuged (14,000 g at 4°C) for 20 min, and the supernatant was then vacuum-dried and re-dissolved in 100 μL of acetonitrile/water (1:1, v/v) for LC-MS/MS analysis using a UHPLC (1290 Infinity LC, Agilent Technologies) coupled to a QTRAP (AB Sciex 5500).

#### LC-MS/MS analysis

Samples were analyzed using an ACQUITY UPLC BEH Amide column (2.1 × 100 mm, 1.7 μm, Waters MS Technologies, Manchester, UK). Mobile phase A was 15 mM CH_3_COONH_4_ in water and mobile phase B was acetonitrile. The column temperature was maintained at 45°C. The flow rate was 400 µL/min, and a 4 µL aliquot of each sample was injected. The gradient was linearly decreased from 90 to 40% B in 0-18 min, increased to 90% in 0.1 min, decreased to 85% in 0.1 min, and then maintained at that level for 18.1-23 min. Quality-control samples, which were prepared from the pooled samples, were injected into the column after every five samples in the analysis sequence to monitor precision and stability.

Conditions in the electrospray ionization-negative mode were set as follows: source temperature, 450°C; ion source gas 1 (Gas1): 45; ion source gas 2 (Gas2): 45; curtain gas (CUR): 30; ion sapary voltage floating (ISVF) -4500 V; and ion pair detection, multiple reaction monitoring (MRM) mode.

#### Data processing

Chromatographic peak area and retention time were extracted in Multi-Quant. Metabolites were identified using the AA standard retention time [17]. To identify differentially expressed metabolites between sample groups, Student’s t-tests were performed; p < 0.05 was considered significantly different.

Boxplots were generated using the quartile calculation method. This involved drawing multiple sets of data on the same coordinate plane to identify distribution differences and changes in expression trends for each data set.

### Western blotting

Mice were sacrificed after neurological behavior testing, and the brain tissue was quickly removed and stored at -80°C. Proteins were extracted from N2a cells after being treated with NaCl medium (40 mM) for 24 h. In detail, the extracted proteins were quantified using a BCA kit (Beyotime, China). Cell lysates and brain tissue of C57BL/6 mice (n=4/group) were homogenized in radioimmunoprecipitation assay lysis buffer containing phosphatase and protease inhibitors (cat. P1049, Beyotime, Shanghai, China). Extracted total protein was determined using the BCA Protein Assay Kit (Beyotime Biotechnology, Shanghai, China). A total of 50 μg protein was loaded onto a 10% or 12% SDS-PAGE gel and subjected to electrophoresis; the separated proteins were then transferred to polyvinylidene fluoride membranes (Bio-Rad, Hercules, CA, USA). Membranes were blocked in TBST containing 5% skim milk for 2 h at room temperature, and then incubated at 4°C overnight with the following primary antibodies: malate dehydrogenase 2 (MDH2, 1:1000, cat. 15462-1-AP, Proteintech Group, Inc.); citrate synthase (CS, 1:5000, cat. 16131-1-AP, Proteintech Group, Inc.); aconitase 2 (ACO2, 1:1000, cat. 11134-1-AP, Proteintech Group, Inc.); fumarate hydratase (FH, 1:1000, cat. 11375-1-AP, Proteintech Group, Inc.); oxoglutarate dehydrogenase (OGDH, 1:5000, cat. 15212-1-AP, Proteintech Group, Inc.); isocitrate dehydrogenase 2 (IDH2, 1:1000, cat. 15932-1-AP, Proteintech Group, Inc.); succinate dehydrogenase complex, subunit B (SDHB, 1:10000, cat. 10620-1-AP, Proteintech Group, Inc); succinate-CoA ligase, ADP-forming, beta subunit (SUCLA2, 1:1000, cat. 12627-1-AP, Proteintech Group, Inc.); brain-derived neurotrophic factor (BDNF, 1:1000, cat. 28205-1-AP, Proteintech Group, Inc.); postsynaptic density protein 95 (PSD95, 1:1000, cat. 20665-1-AP, Proteintech Group, Inc); synaptophysin (Syn, 1:40000, cat. 17785-1-AP; Proteintech Group, Inc.); Tau46 (1:1000, cat. #4019, Cell Signaling Technology); phospho-tau Thr205 (1:1000, cat. #49561, Cell Signaling Technology); phospho-tau Thr181 (1:1000, cat. #12885, Cell Signaling Technology); phospho-tau Thr231 (1:1000, cat. #71429, Cell Signaling Technology); phospho-tau Ser404 (1:1000, cat. #20194; Cell Signaling Technology); SQSTM1/p62 (1:1000, cat. #23214; Cell Signaling Technology); Atg5 (1:1000, cat. #12994; Cell Signaling Technology); Atg5 (1:1000, cat. #12994; Cell Signaling Technology); GAPDH (1:1000, cat. AF0006, Beyotime); and β-actin (1:1000; cat. AF5003; Beyotime). After washing with TBST, the membranes were incubated with secondary antibodies (goat anti-rabbit, 1:1000, cat. A0208; goat anti-mouse, 1:1000, cat. A0192; Beyotime, China) for 2 h at room temperature. Protein bands were visualized using Immobilon Western Chemiluminescent HRP Substrate (Millipore Corporation, Billerica, MA, USA) and imaged using an imaging system (Tanon, Shanghai, China). Gray levels per band were analyzed using ImageJ software.

### Immunofluorescence staining

Mice (n=3/group) were anesthetized and then perfused intracardially with 0.1 M phosphate-buffered saline (PBS). After perfusion with 4% paraformaldehyde, the entire brain was immersed in 4% paraformaldehyde for 24 h and then sectioned into 4 mm paraffin slices using a Rotary Microtome (HM 340E, 9 Thermo Scientific) along the long axis in sagittal plane. After deparaffinization, antigen retrieval was performed in a citric acid solution in a microwave oven (high fire for 5 min, medium fire for 15 min).

For immunostaining analysis, the paraffin sections were blocked with 1.5% normal goat serum at room temperature for 30 min and then incubated with primary antibodies against CS (1:100; cat. 16131-1-AP, Proteintech Group, Inc.) and MDH2 (1:200, cat. ab96193; Abcam) overnight at 4°C. After incubating with secondary antibody (1:300, Alexa Fluor 488-conjugated goat anti-rabbit IgG, cat. ZF-0516, ZSGB-BIO, China) at room temperature for 1 h, the nuclei were stained using DAPI (Beyotime). Coverslips were mounted on the glass slides using Antifade Mounting Medium (Beyotime), and a NEXCOPE microscope (NE900, USA) was used for fluorescence detection.

**Figure 1.**
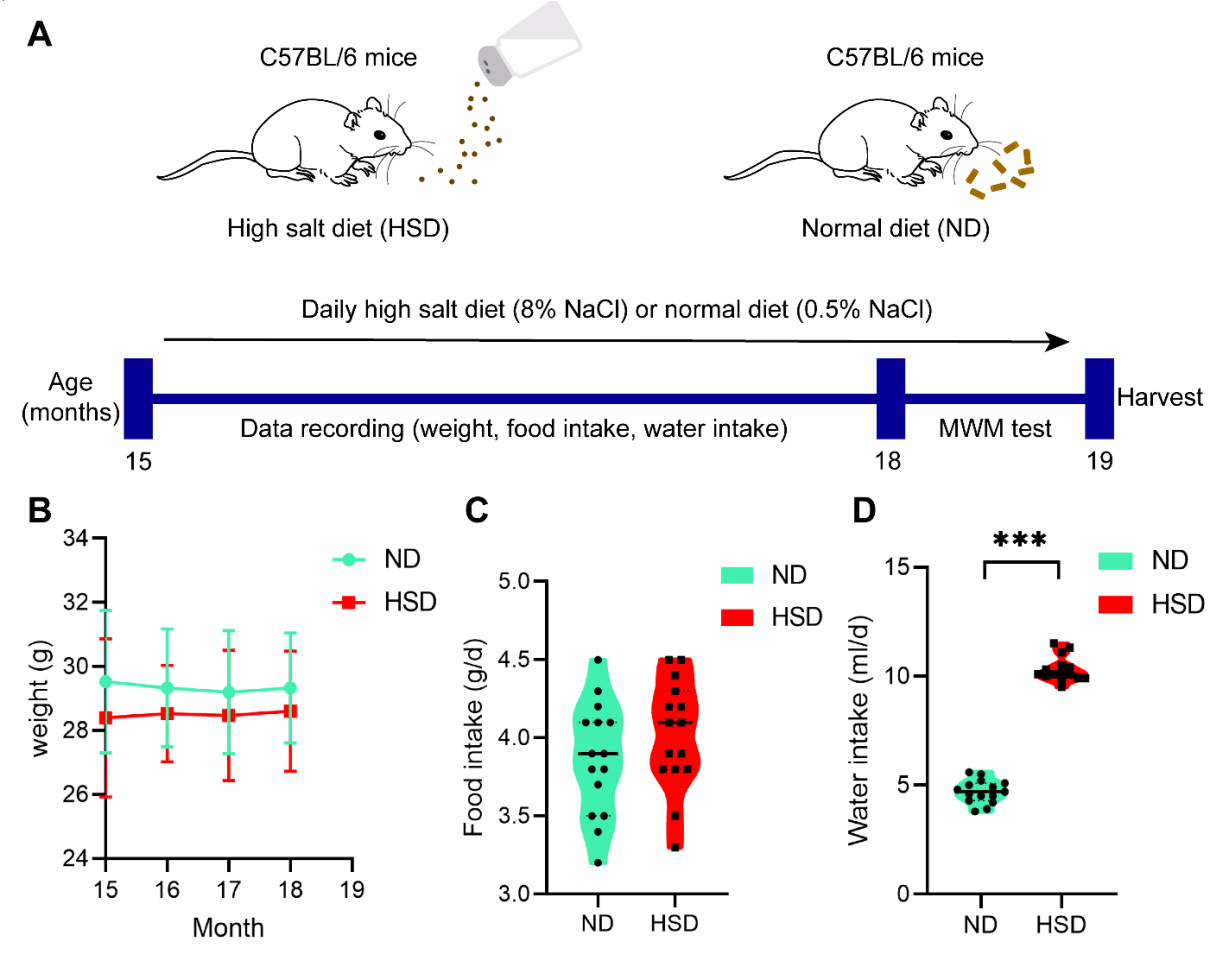
**General characteristics of C57BL/6 mice fed a normal diet or high-salt diet**. C57BL/6 mice (15 months old) were fed HSD or ND for 3 months. (A and B) Alterations in weight and food intake were not significantly different between groups. n=15/group, two-sample t-test. (**C**) HSD group showed higher water intake than that of the ND group. n=15/group, Mann-Whitney U test. mean ± SEM, ***p < 0.001. Abbreviations: HSD: high-salt diet; ND: normal diet.

### TEM

After the MWM, mice (n=3/group) were anesthetized by cervical dislocation. The hippocampus was isolated and cut into ~1 mm^3^ cubes. Specimens were fixed via immersion in 4% buffered glutaraldehyde for 4 h and then washed in cold 1/15 M PBS for 15-30 min three times. Tissues were post-fixed for 2 h in 1% osmium tetroxide (GP18456, Leica) and washed three times in cold 1/15 M PBS for 30 min each. Samples were dehydrated in ascending grades of acetone (50%, 70%, 80%, and 90%, respectively, 15 min each), and then in 100% acetone for 15 min, two times each. Dehydrated tissues were infiltrated for 3 h in embedding medium at room temperature, 60 min in a mixture of 100% acetone/embedding medium (1:1) at 37°C, overnight in a mixture of 100% acetone/embedding medium (1:3) at 37°C, and 5 h in embedding medium in 37°C. All tissues were cured in a 37°C oven for 24 h, followed by 48 h in a 60°C oven to form a solid embedding block, which was sliced into 50 nm sections using an ultramicrotome (EM UC7, Leica). Sections were stained with uranyl acetate for 45 min and then with lead citrate for 15 min. TEM (JEM-1400FLASH, Japan) was used to observe the neurons. Synapse was identified by the presence of synaptic vesicles and postsynaptic densities.

### Statistical analysis

Mouse randomization was based on the random number generator function (RANDBETWEEN) in Microsoft Excel. All experimental data were analyzed using GraphPad Prism version 8.3.0. Shapiro-Wilk test was used to test the normal distribution of the data. Based on the results of the normality test, Student’s *t*-test or Mann-Whitney U test were applied to analyze between-group differences. Data are expressed as means ± standard error of the mean. Significance was set at *p* < 0.05.

**Figure 2.**
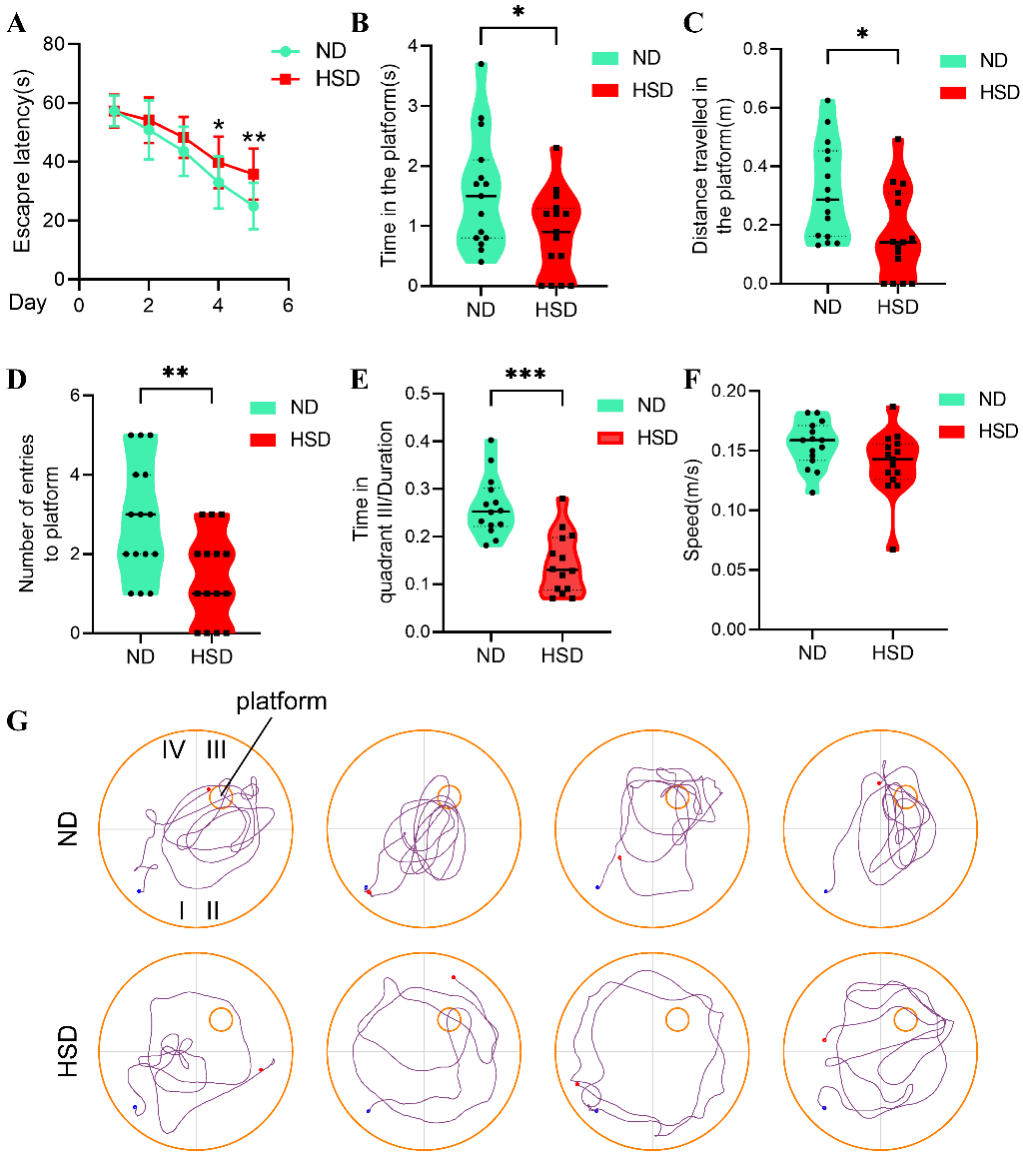
**Dietary salt impairs spatial learning and memory in aged C57BL/6 mice**. Experiment and timeline procedure. Eighteen-month-old C57BL/6 mice were divided into ND and HSD groups. HSD led to significant changes in average escape latency (A), time on the platform (B), distance traveled in the platform (C), number of entries to platform (D), and frequency in the targeted quadrant (E) during the whole trial compared with those of ND. There was no significant difference in the swimming speed between ND and HSD groups. Swimming traces of mice in the probe trial are presented in (G). n=15/group, two-sample t-test. mean ± SEM, *p < 0.05, **p < 0.01, ***p < 0.001 compared with ND group. Abbreviations: HSD: high-salt diet; ND: normal diet.

## RESULTS

### Dietary salt aggravated cognitive impairment in aged C57BL/6 mice

During the 3-month HSD period, we found no significant difference in weight alteration and food intake between the groups (Fig. 1A and B). However, C57BL/6 mice fed HSD (8% NaCl) had higher water intake than that of ND mice (0.4% NaCl) (Fig. 1C).

**Figure 3.**
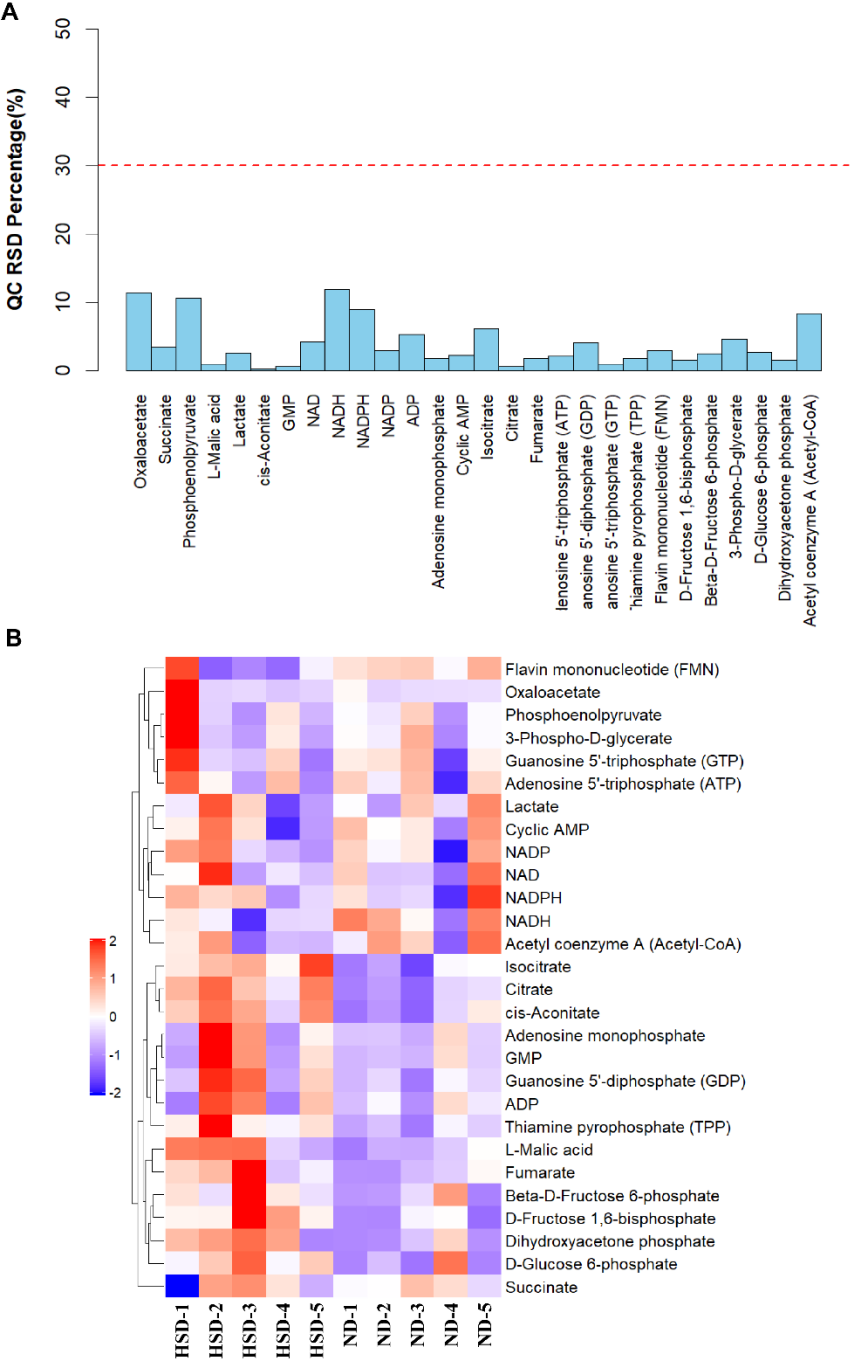
**Relative standard deviation and heatmap of 32 metabolites in glucose metabolism**. (**A**) RSD percentage of all QC samples was less than 12%. (**B**) Heatmap of 32 metabolites in the brain of mice (n=5/group) treated with HSD or ND. Abbreviations: HSD: high-salt diet; ND: normal diet; RSD, relative standard deviation.

Results of MWM revealed that the two groups showed a time-dependent decrease in escape latency after consecutive training. After high-salt administration for 3 months, the HSD group had a longer escape latency than that of the ND group (days 4 and 5, respectively, *p* < 0.05, *p* < 0.01) (Fig. 2A). The HSD group also differed significantly from the ND group in time on the platform, distance traveled to the platform, and number of times entering the platform quadrant during probe trials (Fig. 2B-D). In addition, HSD mice spent less time than ND mice in the targeted quadrant (quadrant III) (Fig. 2E). Swimming speed did not differ between the groups (Fig. 2F), excluding the possibility that the group difference in MWM success was due to a difference in swimming ability. Trajectories of HSD and ND mice during the space exploration task are visualized in Fig. 2G. Overall, our results indicate that chronic HSD exacerbates the impairment of spatial learning and memory in physiologically aged mice.

**Figure 4.**
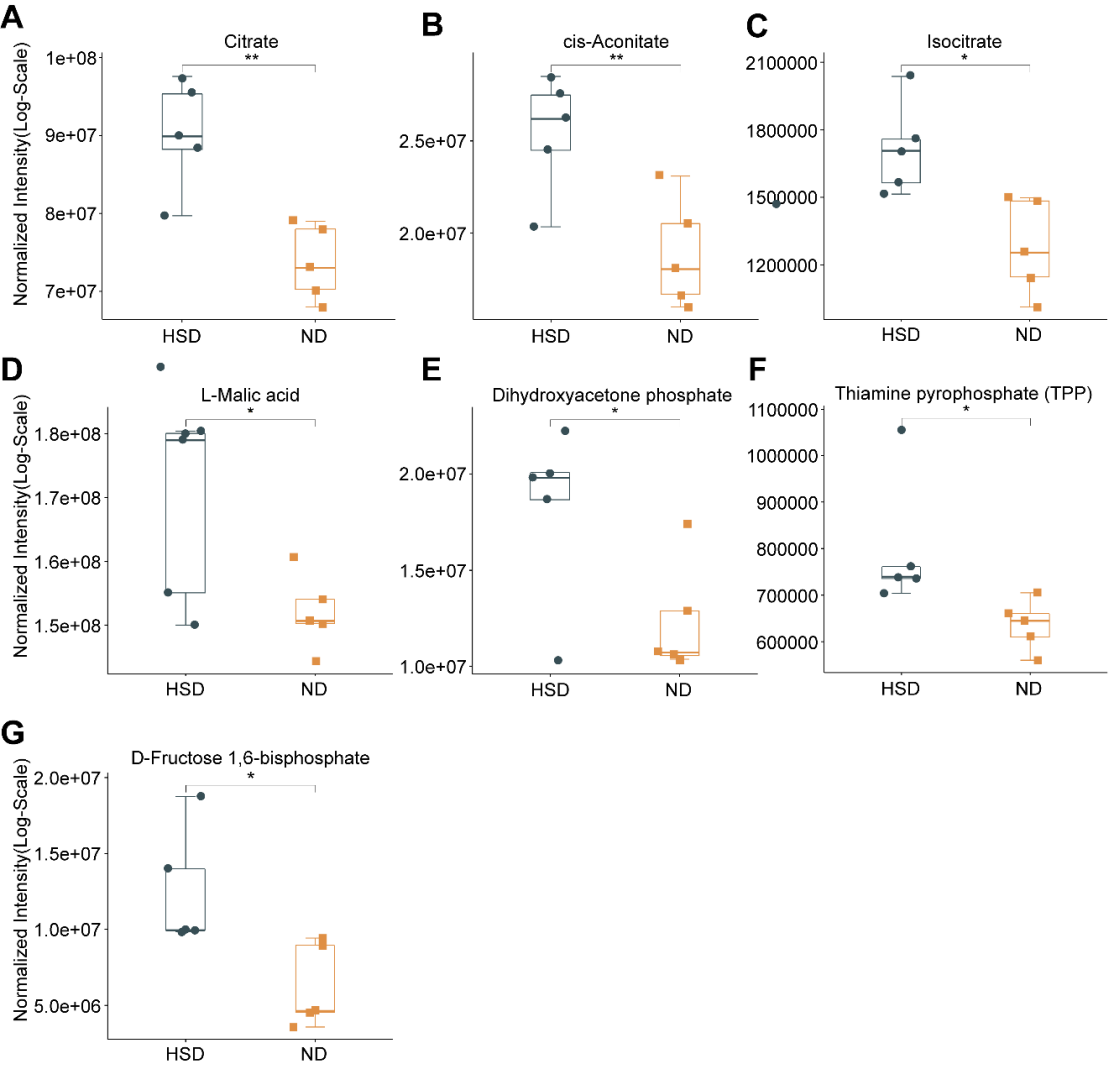
**Boxplot of differences in metabolites between groups**. The levels of citrate, cis-aconitate, isocitrate, and L-malic acid in the TCA cycle, and TPP, DHAP, and D-fructose 1,6-bisphosphate in glycolysis were significantly elevated (n=5/group, using two-sample t-test in citrate, isocitrate, D-fructose 1,6-bisphosphate, DHAP; using Mann-Whitney U test in cis-aconitate, L-malic acid and TPP). Data are presented as the mean ± SEM, *p < 0.05, **p < 0.01. Abbreviations: DHAP: dihydroxyacetone phosphate; F1,6P: D-fructose 1,6-bisphosphate; HSD: high-salt diet; ND: normal diet; TCA: tricarboxylic acid; TPP: thiamine pyrophosphate.

### Glycolysis and the TCA cycle were disturbed in HSD mice

To study the global effects of dietary salt on glucose metabolism, we performed unbiased metabolomic profiling in aged C57BL/6 mice fed an HSD or ND for 3 months (n = 5). Precision, expressed as relative standard deviation, was <12% for all quality control samples (Fig. 3A). A heatmap was used to visually display the difference in the levels of metabolites in glycolysis and the TCA cycle. Of 32 metabolites identified by LC-MS/MS, the expression of seven was significantly higher in the HSD group than in the ND group (Fig. 3B) (*p* < 0.05).

The significantly different metabolites identified between the HSD and ND groups are presented in a boxplot (Fig. 4). Among these seven metabolites, citrate, cis-aconitate, isocitrate, and L-malic acid are part of the TCA cycle, whereas thiamine pyrophosphate (TPP), dihydroxyacetone phosphate (DHAP), and D-fructose 1,6-bisphosphate (F1,6P) are involved in glycolysis. The expression of metabolites associated with glucose metabolism was higher in the HSD group than in the ND group (Fig. 4).

**Figure 5.**
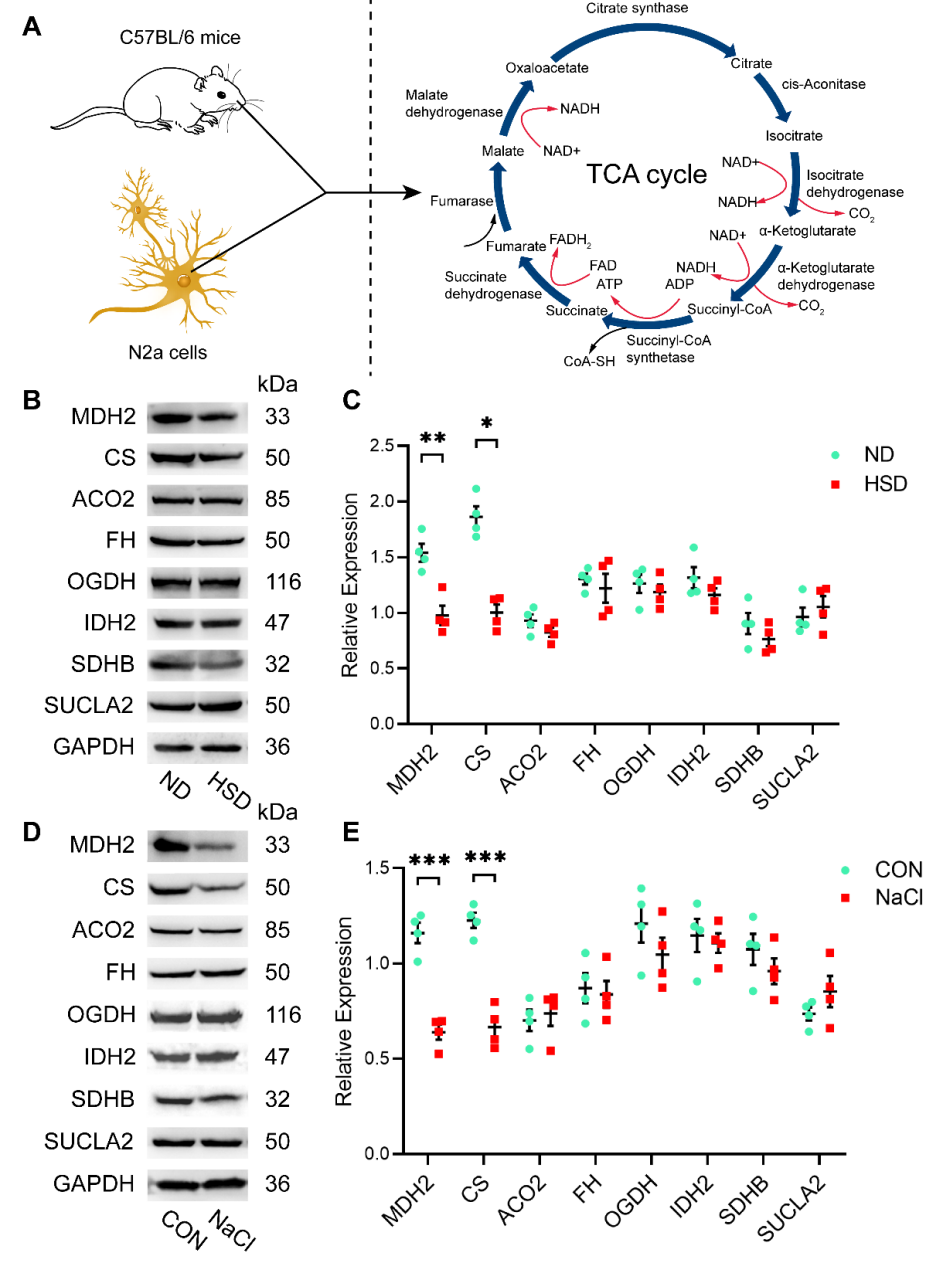
**Expression of related TCA cycle enzymes using western blotting**. (**A**) Diagrammatic sketch of the TCA cycle. (B and C) Western blot results showing that dietary salt decreased the expression of MDH2 and CS in the brain of aged C57BL/6 mice (n=4/group, two-sample t-test). (D and E) MDH2 and CS expression was significantly reduced in N2a cells treated with NaCl medium (40 mM; n=4/group, two-sample t-test). Relative expression of proteins normalized to GAPDH. Data are presented as the mean ± SEM, *p < 0.05, **p < 0.01, ***p < 0.001. Abbreviations: ACO2: aconitase 2; CON: control; CS: citrate synthase; FH: fumarate hydratase; HSD: high-salt diet; IDH2: isocitrate dehydrogenase 2; MDH2: malate dehydrogenase 2; ND: normal diet; OGDH: oxoglutarate dehydrogenase; SDHB: succinate dehydrogenase complex, subunit B; SUCLA2: succinate-CoA ligase, ADP-forming, beta subunit; TCA: tricarboxylic acid.

**Figure 6.**
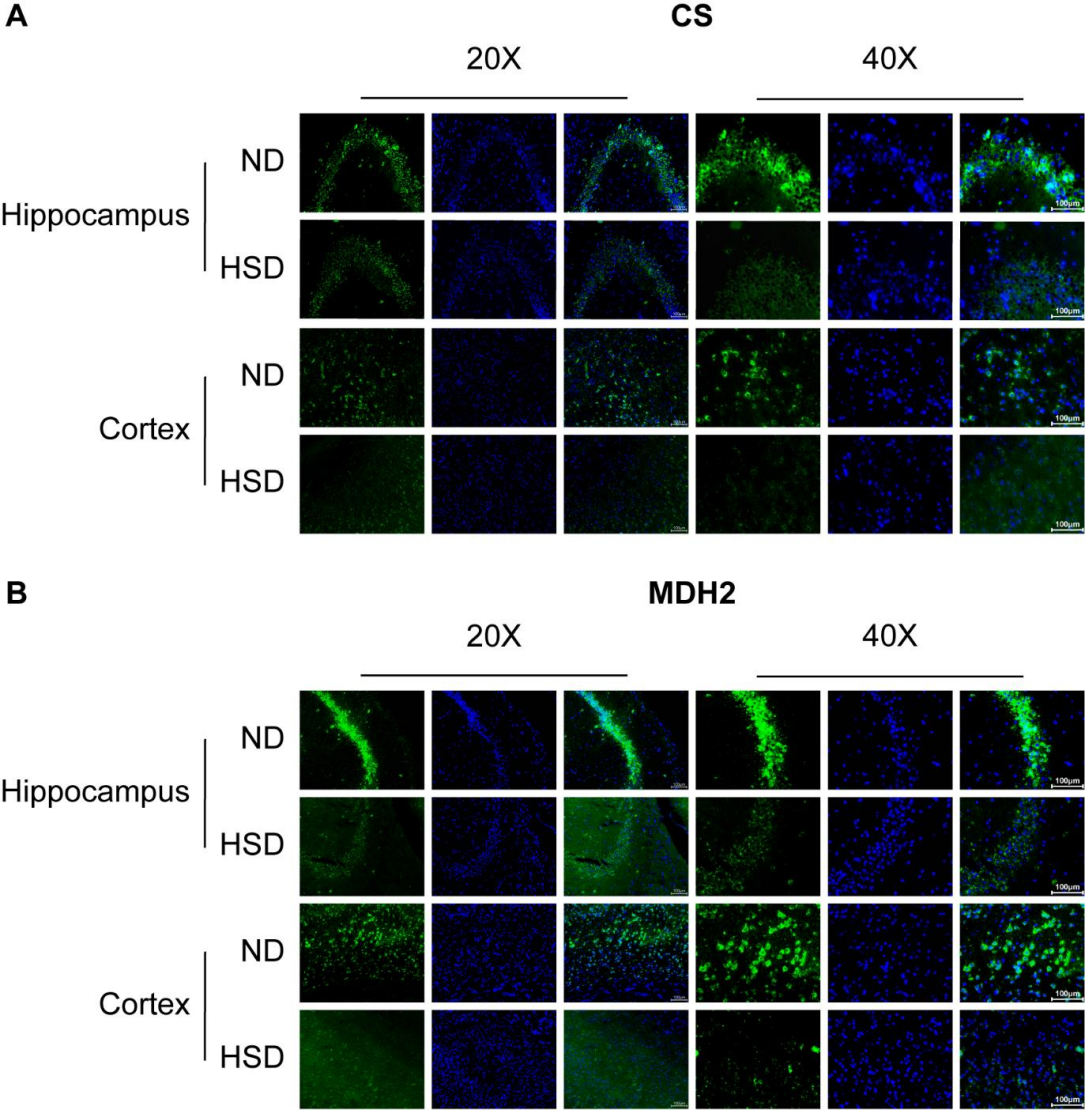
**Expression of related enzymes detected by immunofluorescence staining**. (A and B) CS and MDH2 expression in the hippocampus and cortex observed by immunofluorescence (n=3/group). Scale bars = 100 μm. Abbreviations: CON: control; CS: citrate synthase; HSD: high-salt diet; MDH2: malate dehydrogenase 2; ND: normal diet.

We detected all TCA cycle proteins using western blotting in both animal and cell models (Fig. 5A). Western blots provided evidence that CS and MDH2 protein expression was gradually downregulated in the HSD group compared with that in the ND group, but that of ACO2, IDH2, OGDH, SDHB, and SUCLA did not change significantly (Fig. 5B and C). Our in vitro HSD model using N2a cells was in line with the in vivo results. Similarly, western blots showed that MDH2 and CS expression was significantly lower in the NaCl group than in the CON group in N2a cells. The expression of other TCA enzymes, including ACO2, FH, IDH2, OGDH, SDHB, and SUCLA, did not differ between the groups (Fig. 5D and E). Thus, the western blotting results are in agreement with the LC-MS/MS results in both aged C57BL/6 mice and N2a cells.

Additionally, the immunofluorescence results indicated that the fluorescence intensity of both CS and MDH2 proteins was lower in the HSD group than in the ND group in the hippocampus and cortex (Fig. 6A and B), confirming the western blotting results. Thus, TCA enzyme expression differed in response to dietary salt.

**Figure 7.**
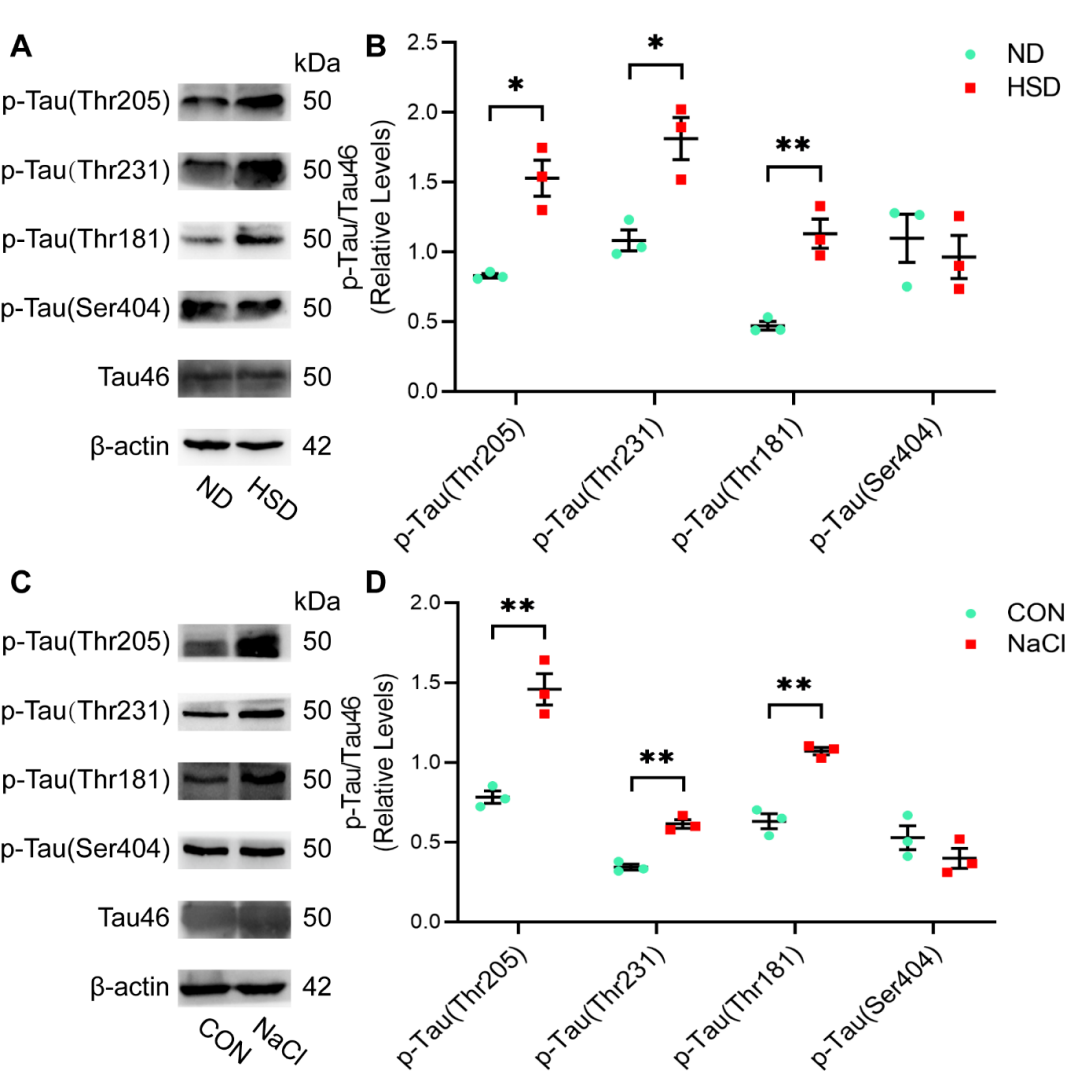
**Long-term dietary salt induces tau hyperphosphorylation**. (A and B) Three months of high salt treatment led to p-Tau (Thr205, Thr231, and Thr181) without increasing total tau levels (tau46) in aged C57BL/6 mice (n=3/group, two-sample t-test). (C and D) N2a cells treated with NaCl medium (40 mM) showed increased expression of p-Tau (Thr205, Thr231, and Thr181) compared with that of the CON group (n=3/group, two-sample t-test). Relative expression of proteins normalized to β-actin. Data are expressed as the mean ± SEM, *p < 0.05. **p < 0.01. Abbreviations: CON: control; HSD: high-salt diet; ND: normal diet.

### High salt diet caused tau hyperphosphorylation in aged C57BL/6 mice and N2a cells

We used western blotting to analyze the expression of p-tau and total tau levels (detected using Tau46). The HSD group showed a prominent increase in phosphorylated tau (Thr205, Thr231, and Thr181) without a corresponding increase in total tau levels after 3 months of salt intake. In contrast, p-tau (Ser404) levels were not significantly elevated in the HSD group (Fig. 7A and B). These patterns were also present in N2a cells treated with NaCl (Fig. 7C and D).

### Dietary salt induced neuron damage and synapse loss

**Figure 8.**
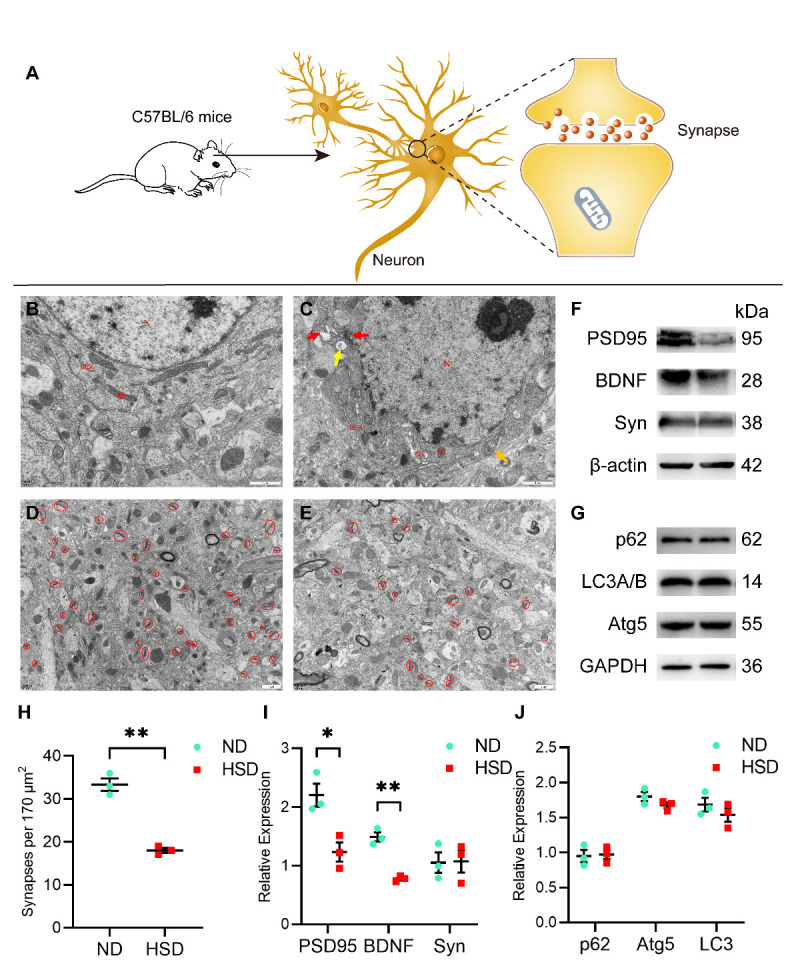
**Ultrastructure of neurons and synapses**. (**A**) Diagrammatic sketch of synapse. (**B**) In the ND group, the neurons were intact with elliptic nuclei, evenly distributed chromatin, and intact nuclear membrane. Mitochondria, rough endoplasmic reticulum, and ribosomes were intact and clear. (**C**) In the HSD group, the neuronal cell bodies were slightly wrinkled with aggregated chromatin in the nuclei and intact nuclear membranes. Electron density in the cytoplasm was increased, and a few mitochondria showed mild swelling (orange arrow). In addition, a small amount of autophagy (red arrow) and vacuoles (yellow arrow) was observed. N: nucleus, Mi: mitochondria, RER: rough endoplasmic reticulum, GB: Golgi body. (D, E, H) Compared with the ND group, the HSD group showed a significantly lower density of synapse (red circle) per 170 μm^2^ by electron microscopy (n=3/group). (F, G, I & J) Western blotting was used to detect the expression of PSD95, BDNF, Syn, p62, Atg5, and LC3. Relative expression of proteins normalized to β-actin and GAPDH (n=3/group, two-sample t-test). Data are expressed as the mean ± SEM; *p < 0.05, **p < 0.01. Scale bar = 1 μm. Abbreviations: BDNF: brain-derived neurotrophic factor; HSD: high-salt diet; ND: normal diet; PSD95: postsynaptic density protein 95; Syn: synaptophysin.

A schematic of the neuron synapse in C57BL/6 mice is shown in Figure 8A. TEM observations revealed that neurons were intact in the ND group, with elliptical nuclei, evenly distributed chromatin, and an intact nuclear membrane. In the cytoplasm, mitochondria, rough endoplasmic reticulum, and ribosomes were intact and clear (Fig. 8B). In the HSD group, however, neurons tended to undergo apoptosis. Cell bodies were slightly wrinkled with aggregated chromatin in the nucleus, although the nuclear membrane was intact. The electron density increased in the cytoplasm, and a few mitochondria showed mild swelling. We also observed small amounts of autophagy and vacuoles (Fig. 8C).

Dietary salt for 3 months caused significantly more synaptic loss in the brains of aged C57BL/6 mice than that of a ND (Figure 8D, E, and H). In addition, we investigated the effect of HSD on synapse-associated proteins and BDNF using western blotting. Aged HSD mice exhibited lower PSD95 and BDNF expression than mice in the ND group, whereas Syn levels did not change significantly between the groups (Figure 8F and I). However, the expression of key autophagy proteins, including p62, Atg5, and LC3, did not decrease significantly in the HSD group compared with that in the ND group (Figure 8G and J).

The combined results from TEM and western blotting demonstrated that long-term salt intake in aged C57BL/6 mice leads to synaptic dysfunction and neuronal damage. In turn, spatial memory and learning capacity are impaired, consistent with the MWM results.

**Figure 9.**
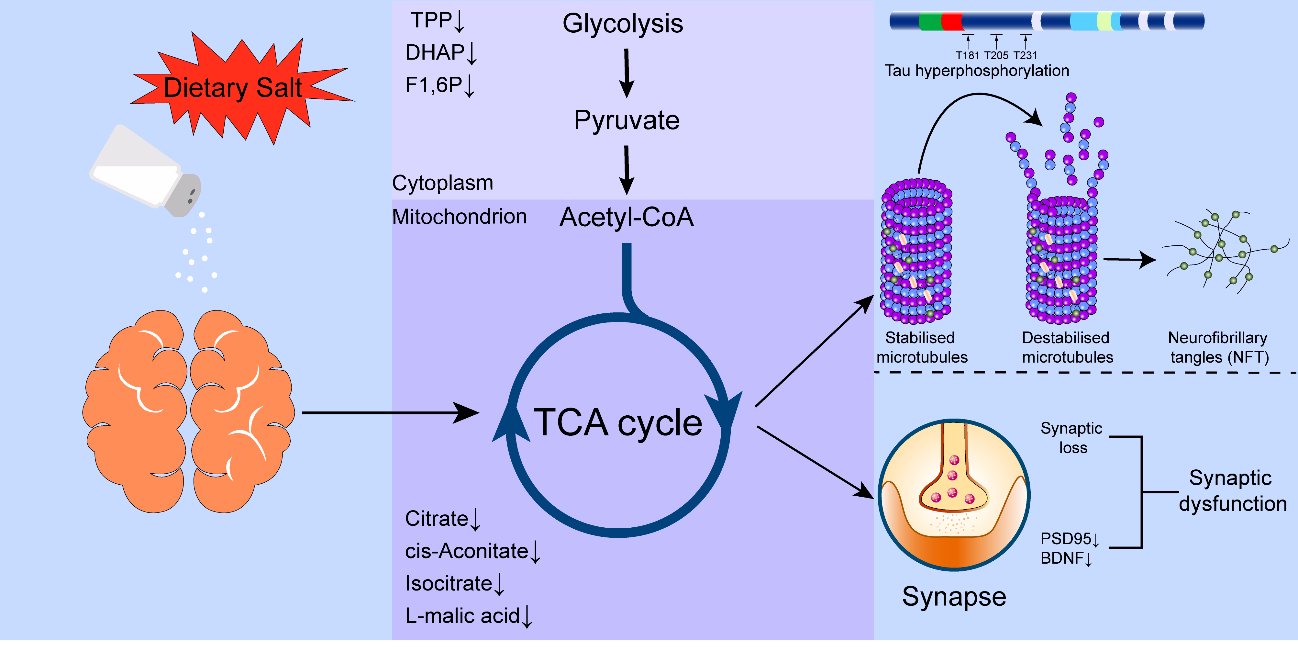
**Potential mechanism of salt-induced tau hyperphosphorylation and synaptic impairment**. Long-term exposure to high dietary salt led to disturbed glycolysis in the cytoplasm and a “broken” TCA cycle in the mitochondria. The subsequent energy metabolism disorder caused tau hyperphosphorylation at multiple sites and synaptic deficits, which ultimately results in cognitive impairment. Abbreviations: BDNF: brain-derived neurotrophic factor; DHAP: dihydroxyacetone phosphate; F1:6P: D-fructose 1,6-bisphosphate; PSD95: postsynaptic density protein 95; TCA: tricarboxylic acid, TPP thiamine pyrophosphate.

## DISCUSSION

Dietary salt plays a neurotoxic role in the pathogenesis of cognitive impairment via the promotion of tau hyperphosphorylation [6] and suppression of synaptic protein expression [7]. In this study, we found that a long-term HSD significantly altered water intake but not body weight or food intake. Additionally, we showed that aged C57BL/6 mice on HSD had a longer escape latency during MWM than that of control mice. The two groups also differed in time spent on the platform, distance traveled toward the platform, number of entries onto the platform, and time spent in the targeted quadrant. Thus, long-term dietary salt impaired spatial memory and learning ability in aged mice. This effect was not a result of changes in swimming ability, as HSD had no impact on that trait.

In addition, we investigated the effect of HSD on tau phosphorylation and synaptic function in the brains of aged C57BL/6 mice and NaCl-treated cell models. Our results indicated that dietary habits undoubtedly promote tau hyperphosphorylation at multiple genomic sites in mice, and the cell model results confirmed these in vivo findings. Hyperphosphorylated tau indirectly drives tau aggregation, which then sequester other cell components and cause cognitive deficits [18]. In terms of synapse function, TEM results showed that 3 months of HSD caused neural impairment and synaptic loss in aged C57BL/6 mice. Synaptic loss is the major cause of cognitive impairment in Alzheimer’s disease [19, 20]. We also observed significant reductions to PSD95 and BDNF levels in the brains of HSD mice, with inhibition of both proteins linked to impaired synaptic plasticity and the associated negative effects on learning and memory [21, 22]. Our results are in line with those of these recent studies suggesting that tau plays a pathological role in synaptic decline. Abnormal tau phosphorylation impairs the transport of glutamate receptor subunits (e.g., GluA1, GLUA2/3, and NR1) to the postsynaptic density [23, 24], eventually leading to synapse loss from reduced mitochondria-dependent ATP production [25]. However, our results do not clarify the exact mechanism linking tau phosphorylation with impairments in synaptic function.

Although it remains to be fully understood how dietary salt induces tau hyperphosphorylation and synaptic dysfunction, we established that a “broken” TCA cycle may be associated with HSD-triggered memory impairment. Our LC-MS/MS and western blot analysis of glycolysis intermediates and key enzymes in the TCA cycle showed significant differences between the HSD and control groups. Unexpectedly, we did not observe significant between-group variation in ATP levels. We conjecture that the compensatory capacity of the TCA cycle allows other pathways to introduce metabolites and maintain normal energy production. However, we may observe lower ATP with a longer treatment duration because dietary salt has a cumulative effect on the brain over time. Both our in vivo and in vitro results showed that high salt intake reduced MDH2 and CS expression and fluorescence intensity in the cortex and hippocampus of aged mice. These results are all in line with those of previous research showing how elevated salt intake decreases the activity of key enzymes in the TCA cycle [14]. Previous studies have suggested that dietary salt restriction ameliorates insulin resistance, both in a rat model of metabolic syndrome [26] and in Dahl salt-sensitive rats [27]. Moreover, reductive TCA cycle flux can regulate insulin secretion via the production of stimulus-secretion coupling factors [28]. Brain insulin resistance has been reported to contribute to tau hyperphosphorylation and cognitive impairment [29]. Nevertheless, whether high salt intake affects tau hyperphosphorylation and synaptic deficits through the TCA cycle still needs further verification. Future studies should follow up on promising findings regarding the association among the TCA cycle, tau hyperphosphorylation, and synapse-related proteins [12-14].

In conclusion, we demonstrated for the first time that long-term HSD disturbed the TCA cycle in the brains of aged mice. Our results provide a novel insight into the potential mechanisms of salt-induced tau hyper-phosphorylation and synaptic impairment (Fig. 9). Although the exact mechanisms remain unclear, this study can form the basis of future research examining high-salt-induced tau phosphorylation and synaptic dysfunction from the perspective of energy metabolism. Investigations in this vein could provide considerable benefits to the development of therapy for cognitive disfunction.
